# Clinical Significance and Potential Molecular Mechanisms of Angiotensin-Converting Enzyme 2 in Colorectal Cancer

**DOI:** 10.14740/wjon2650

**Published:** 2025-12-17

**Authors:** Da Tong Zeng, Li Yang, Jia Ying Wen, Ke Jun Wu, Guo Qiang Chen, Zong Yu Li, Jing Wen Ling, Bei Bei Huang, Ying Yi Xie, Yi Yu Dong, Ye Ying Fang, Dan Ming Wei, Gang Chen, Lin Shi, Wei Jian Huang

**Affiliations:** a Department of Pathology, Redcross Hospital of Yulin City, Yulin, Guangxi Zhuang Autonomous Region 537000, China; b Department of Pathology, The Second Affiliated Hospital of Guangxi Medical University, Nanning, Guangxi Zhuang Autonomous Region 530000, China; c Department of Radiotherapy, The Second Affiliated Hospital of Guangxi Medical University, Nanning, Guangxi Zhuang Autonomous Region 530000, China; d Department of Pathology, The First Affiliated Hospital of Guangxi Medical University, Nanning, Guangxi Zhuang Autonomous Region 530021, China; e Department of Medical Information Engineering, School of Information and Management, Guangxi Medical University, Nanning, Guangxi Zhuang Autonomous Region 530199, China; f Department of Radiotherapy, The First Affiliated Hospital of Guangxi Medical University, Nanning, Guangxi Zhuang Autonomous Region 530021, China; g These authors contributed equally to this article.

**Keywords:** Angiotensin-converting enzyme 2, Colorectal cancer, Immune microenvironment, Immunosuppressive, Nerve invasion, PD-L1, CD8, Mucinous adenocarcinoma, *NRAS* (Q61R/L/H/K) mutation

## Abstract

**Background:**

Angiotensin-converting enzyme 2 (ACE2) exhibits tumor-suppressive potential in cancers, but its role in colorectal cancer (CRC) is unclear. The aim of the study was to investigate ACE2 expression, clinical significance, and immune microenvironment associations in CRC.

**Methods:**

A multidimensional approach was taken using single-cell RNA sequencing and spatial transcriptomics to analyze ACE2 expression in CRC cells. High-throughput data from the Gene Expression Omnibus (GEO) and The Cancer Genome Atlas (TCGA) (2,275 CRC and 1,269 adjacent tissues) were used to assess mRNA levels. Immunohistochemistry was performed to examine ACE2 protein expression in 66 CRC and 75 adjacent tissues. Molecular testing assessed associations with Kirsten rat sarcoma viral oncogene homolog (*KRAS*), neuroblastoma RAS viral oncogene homolog (*NRAS*), and B-Raf proto-oncogene, serine/threonine kinase (*BRAF*) mutations. Immune infiltration was analyzed using single-sample gene set enrichment analysis (ssGSEA), focusing on 24 immune cell types, CD8^+^ T cells, and programmed death ligand 1 (PD-L1) correlations.

**Results:**

ACE2 was highly expressed in malignant cells and Ki-67-activated regions. mRNA and protein levels were upregulated in CRC (standardized mean difference (SMD) = 0.321, area under the curve (AUC) = 0.844). High ACE2 exhibited significant associations with nerve invasion, lower expression in mucinous adenocarcinomas, and *NRAS* (Q61R/L/H/K) mutations. ACE2 negatively showed an inverse correlation with CD8^+^ T-cell infiltration (r = -0.186, P < 0.001) and PD-L1 expression (r = -0.282, P = 0.022).

**Conclusions:**

The upregulation of ACE2 is associated with nerve invasion, pathological type, and an immunosuppressive microenvironment with reduced CD8^+^ T-cell infiltration and PD-L1 expression.

## Introduction

### Background

Cancer is garnering increased attention as a prominent cause of mortality [1-4]. In 2020, colorectal cancer (CRC) was responsible for over 1.9 million newly diagnosed cases and 935,000 deaths, representing approximately 1 in 10 cancer cases and fatalities and ranking third in incidence and second in mortality [5]. The global economic impact of cancer is staggering, with a projected financial burden of $25.2 trillion on the world economy from 2020 to 2050. Among various types of cancer, CRC stands out as the second-highest contributor to this cost [6]. Moreover, research on the pan-cancer burden and the socioeconomic development index (SDI) has shown that a higher SDI is often associated with an increased risk of CRC [7]. CRC is a multifaceted, intricate disease with a large number of factors contributing to its pathogenesis, including genetic mutations, abnormalities in signaling pathways, DNA methylation, epigenetic regulation, inflammatory response, and immune regulation [8-14]. These factors interact with one another to promote the occurrence and development of CRC. In patients with tumors, both protumor and antitumor factors are induced in the body, including increased neutrophil and platelet levels and decreased lymphocyte levels [15, 16]. A comprehensive understanding of these mechanisms is of paramount significance to elucidate the pathophysiological processes associated with CRC, facilitate the development of innovative therapeutic strategies, and enable accurate prognostic assessments [17].

Oncology patients exhibit heightened susceptibility to severe acute respiratory syndrome coronavirus 2 (SARS-CoV-2) infection and are at an elevated risk of experiencing critical events during the course of a novel coronavirus pneumonia pandemic, such as the coronavirus disease 2019 (COVID-19) pandemic [18]. Angiotensin-converting enzyme 2 (ACE2), which serves as the primary receptor for host cell entry [19-21], has drawn significant attention in scientific circles. ACE2 is not only a monocarboxypeptidase but also a functional receptor located on the cell surface [22]. It plays a pivotal role in regulating the renin-angiotensin system (RAS) by catalyzing the hydrolysis of angiotensin (Ang) II into Ang-(1-7), thereby inhibiting angiotensin II type 1 receptor (AT1R) activation and exerting vasoconstrictive, proinflammatory, and thrombotic effects. Additionally, Ang-(1-7) stimulates the release of nitric oxide, prostaglandin E2, and bradykinin through Mas receptors, leading to vasodilation, natriuresis, and attenuation of oxidative stress and inflammation [23, 24].

ACE2 exhibits significant expressions not only in the heart, kidney, and respiratory tract but also in the colon [25-27]. In the context of cancer, ACE2 shows an inverse correlation with the activation of several oncogenic pathways, such as cell cycle, transforming growth factor-beta (TGF-β), Wnt, and vascular endothelial growth factor (VEGF), thereby impeding tumor proliferation and epithelial-mesenchymal transition (EMT) [28-30]. Furthermore, ACE2 is positively associated with antitumor immune response and serves as a favorable prognostic indicator in diverse cancer types, implying a potential protective function of ACE2 in cancer progression [31]. Previous investigations have indicated the potential involvement of the RAS in the pathophysiology of CRC initiation and progression [32-34]. Moreover, it has been proposed that ACE2 and its associated factors contribute to the underlying mechanisms linking CRC and COVID-19 [35]. Notably, several studies have confirmed the heightened expression of the ACE2 protein in human CRC [36]. Nevertheless, the available literature on ACE2 expression and its clinical significance in CRC remains scarce, and the extant reports have limitations, including small sample sizes, an absence of validation using independent clinical samples, and a dearth of molecular mechanism investigations.

### Aim

In this investigation, we postulate that elevated ACE2 expression is closely associated with colon cancer development. To explore the potential clinical significance and molecular mechanisms of ACE2 in CRC, we conduct in-house experiments encompassing immunohistochemistry (IHC), RNA sequencing data collection, genetic testing (B-Raf proto-oncogene, serine/threonine kinase (*BRAF*)-V600E/Kirsten rat sarcoma viral oncogene homolog (*KRAS*)/neuroblastoma RAS viral oncogene homolog (*NRAS*)), and analysis of clinical parameters. Additionally, we seek to gain further insights into the relationship between ACE2 expression and the level of immune cell infiltration in colon cancer by assessing programmed death ligand 1 (PD-L1) and CD8. These findings hold promise for advancing the clinical management of colon cancer and enhancing our understanding of its pathogenesis.

## Materials and Methods

### Single-cell RNA (scRNA) sequencing analysis of ACE2 in CRC cells

For scRNA sequencing (scRNA-seq) analysis of ACE2, we used the cancer-related GSE201348 scRNA dataset, publicly released on April 28, 2022, and accessible through the Gene Expression Omnibus (GEO) database. First, data preprocessing and filtering were performed using the Seurat package. Genes expressed in fewer than three cells and cells expressing fewer than 50 genes were removed. Next, during the quality control process, cells expressing more than 500 genes and with mitochondrial content below 25% were retained. After data normalization, principal component analysis was performed for dimensionality reduction, and the first 20 principal components were selected for clustering analysis. Subsequently, the Uniform Manifold Approximation and Projection (UMAP) algorithm was used for secondary dimensionality reduction. Additionally, the InferCNV package was employed to identify malignant tumors.

### Spatial transcriptome analysis of ACE2 in CRC cells

Spatial transcriptomic data were acquired using the 10x Genomics Visium platform, followed by normalization and preprocessing using Seurat v5. To elucidate the spatial distribution of distinct cell types within tissue, deconvolution analysis was applied to map single-cell transcriptomic reference data onto spatial transcriptomic data. This allowed for the estimation of the relative compositional proportions of epithelial, stromal, and immune cells at each spatial spot. Based on the deconvolution results, three cell-type-specific scores (epithelial score, stromal score, and immune score) were calculated. The cell type corresponding to the highest score was defined as the dominant cell type for that spatial spot. Subsequently, spatial distribution maps of these three scores were visualized on the spatial coordinates of tissue sections to reflect the histological localization of different cell populations. Additionally, the spatial expression profiles of key genes - *MKI67* (a cell proliferation marker) and *ACE2* (a receptor gene) - were analyzed. Approximate IHC H-scores were computed based on the distribution of expression levels. Four intensity grades (0, 1+, 2+, and 3+) were defined using expression quantiles, and H-scores for each cell-type-enriched region were calculated using the formula H = 1 × %1 + 2 × %2 + 3 × %3. These scores were used to compare ACE2 expression among distinct cell types.

### Collection and processing of datasets

To ensure comprehensive data retrieval, high-throughput datasets were systematically queried against multiple databases, including the Gene Expression Omnibus, ArrayExpress [37], the Sequence Read Archive, Oncomine, The Cancer Genome Atlas (TCGA), and PubMed. Relevant mRNA microarray data were extracted, encompassing a comprehensive cohort of 2,275 CRC samples and 1,269 adjacent samples. The search strategies employed were based on the keywords “colorectal cancer” or “CRC” and “mRNA” or “gene.” In the case of mRNA microarrays, the inclusion criteria were defined as follows: 1) the presence of diagnosed CRC tissue in the samples; 2) each chip containing both CRC and corresponding normal tissues; 3) the availability of ACE2 expression data; and 4) the samples originating from *Homo sapiens*. The exclusion criteria were as follows: 1) an absence of ACE2 expression data; 2) a lack of normal controls; and 3) samples originating from non-*Homo sapiens* species. To augment the normal samples and enhance the dataset, the Genotype Expression (GTEx) project was used in combination with the TCGA. The detailed datasets and sample sizes are shown in Table 1.

**Table 1:** The Datasets Used in the Research

Study	Non-CRC	CRC
GPL10558	129	89
GPL15207	23	37
GPL570	239	645
GPL6480	14	206
GPL96	104	268
GSE103512	12	57
GSE113513	14	14
GSE115261	10	10
GSE141174	3	3
GSE156355	6	6
GSE15781	20	22
GSE20842	65	65
GSE25071	4	46
GSE28000	6	5
GSE41011	12	19
GSE44076	98	98
GSE47063	4	14
GSE87211	160	203
TCGA_GTEx	349	471

CRC: colorectal cancer; TCGA: The Cancer Genome Atlas; GTEx: Genotype Expression.

The gene expression values from the publicly available datasets underwent a log_2_(X + 1) transformation, where X represents the raw data of gene expression. Microarrays from the Gene Expression Omnibus database with the same platform were merged to facilitate subsequent analysis. To mitigate batch effects within the combined microarrays, we employed the surrogate variable analysis package in R (v. 4.0.2). Subsequently, ACE2 expression was quantified based on the aforementioned datasets.

An integrated diagnostic performance analysis of 19 studies showed that the pooled sensitivity was moderate, and the pooled specificity was moderate to high, with all studies consistent with the overall estimate. Deeks’/Egger’s funnel plots did not indicate significant small-study effects or publication bias. After sensitivity analyses using multiple methods, there were no substantial changes in the pooled results, confirming that the pooled estimate of diagnostic accuracy in this study was robust.

### Expression of the ACE2 protein in CRC

All experiments were performed according to applicable rules. IHC was employed to assess the protein expression of ACE2. A total of 66 CRC and 76 adjacent colon tissue samples were procured for this study from Shanghai Outdo Biotech Company, China (catalog no. HColA160Bc01). The formalin-fixed and paraffin-embedded samples underwent a dehydration procedure, followed by inhibition of endogenous peroxidase activity and antigen retrieval. The two-step IHC technique was employed [38]. An anti-ACE2 antibody (rabbit polyclonal to ACE2, Abcam) was employed as the first antibody in the experimental group, and phosphate-buffered saline was used as a comparison, replacing the initial antibody. Both were incubated overnight at 37 °C, after which the second antibody was added to the samples at 25 °C and incubated for 0.5 h. After hematoxylin staining, two pathologists independently evaluated the staining intensity and the percentage of stained positive cells. The score of ACE2 protein expression was the product of the staining strength score and the score of the percentage of positive stained cells, as previously reported [39].

### Statistical analysis of ACE2 expression in CRC

The ACE2 protein’s differential expression in CRC tissues and non-CRC colorectal tissues was examined using Student’s *t*-test via IBM SPSS Statistics 26 software. Receiver operating characteristic (ROC) curves, generated using GraphPad Prism 8 software, were used to evaluate ACE2’s ability to discriminate between CRC tissues and non-CRC colorectal tissues, with the area under the curve (AUC) serving as a measure of its accuracy. Fisher’s exact test was used to analyze the enumeration data, specifically investigating the associations between ACE2 expression and various clinicopathological parameters, including sex, age, tumor node metastasis stage, pathological stage, pathological type, tumor size, tumor number, nerve invasion, and vascular invasion. These parameters were obtained from IHC samples. Statistical significance was assessed at a threshold of P < 0.05.

To enhance confidence in the findings, we amalgamated high-throughput datasets to augment the sample size. The standardized mean difference (SMD) and summary ROC (SROC) curves were employed as the primary indicators to quantify ACE2 expression and discriminative ability in CRC. Calculations were performed using Stata 15.0 software. The SMD and 95% confidence interval (CI) were employed to compare ACE2 expression between CRC and non-CRC colorectal tissues. The heterogeneity of the pooled-analysis results was evaluated using the I^2^ statistic. A P value of less than 0.05 or an I^2^ exceeding 50% indicated significant heterogeneity, whereupon a random-effects model was employed. Conversely, in cases in which the P value was greater than 0.05 or the I^2^ less than 50%, no significant heterogeneity was detected, allowing for the use of a fixed-effects model. Publication bias was assessed using Egger’s test. SROC curves were generated using Stata software.

### Molecular testing and statistical analysis of ACE2-associated genes

To identify genes closely associated with ACE2 expression, molecular testing of *KRAS*, *NRAS* (G12C/D/S), *NRAS* (Q61R/L/H/K), and *BRAF*-V600E was conducted on in-house samples. The relationship between ACE2 expression and *KRAS*, *NRAS* (G12C/D/S), *NRAS* (Q61R/L/H/K), and *BRAF*-V600E was assessed using Student’s *t*-test and Pearson correlation analysis on SPSS 26.

### Correlation and immune infiltration analysis of ACE2 in CRC

The study analyzed the correlation between the primary variable, ACE2 (ENSG00000130234.12), and the immune infiltration matrix data. Correlation analysis was performed using the Spearman method, and the results were visualized with a lollipop plot using the ggplot2 package in R. Immune infiltration was assessed using the ssGSEA algorithm from the Gene Set Variation Analysis (GSVA) package (version 1.46.0), which calculated the infiltration levels of 24 immune cell types based on marker genes provided by Immunity. The 24 immune cell types included activated dendritic cells (aDCs), B cells, CD8 T cells, cytotoxic cells, dendritic cells (DCs), eosinophils, immature dendritic cells (iDCs), macrophages, mast cells, neutrophils, natural killer (NK) CD56 bright cells, NK CD56 dim cells, NK cells, plasmacytoid dendritic cells (pDCs), T cells, T helper cells, T central memory (Tcm) cells, T effector memory (Tem) cells, T follicular helper (TFH) cells, T gamma delta (Tgd) cells, Th1 cells, Th17 cells, Th2 cells, and regulatory T cells (Tregs). Data were obtained from the TCGA database, specifically from the TCGA-COAD (CRC) project, where RNA-seq data processed via the Spliced Transcripts Alignment to a Reference (STAR) pipeline were downloaded and converted to transcripts per million (TPM) format, along with clinical data. Normal samples were excluded during data filtering, and the data were processed using a log_2_(value + 1) transformation.

### Expression of CD8 and PD-L1 in CRC

To identify immune-related molecules closely associated with the expression of ACE2, an IHC test targeting CD8 and PD-L1 was conducted on the in-house samples. The relationship between ACE2 expression and both CD8 and PD-L1 was examined using Student’s *t*-test and Pearson correlation analysis using SPSS 26.

### Ethical considerations

This research adhered to the fundamental medical ethical principles outlined in the Helsinki Declaration and received approval from the Ethics Committee of Redcross Hospital of Yulin City (contract no. Z-K20221823).

## Results

### Expression of ACE2 in single-cell and spatial transcriptomics

**Figure 1:**
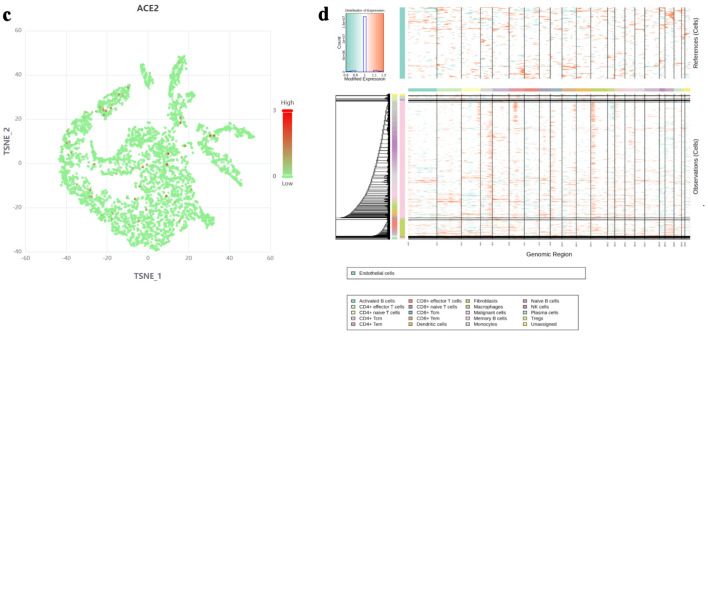
The expression level of ACE2 protein in different cell types based on single-cell RNA sequencing. (a) t-SNE plot showing the distribution of various cell types (activated B cells, fibroblasts, CD4^+^ naive T cells, macrophages, CD4^+^ effector T cells, malignant cells, memory B cells, monocytes, naive B cells, plasma cells, Tregs, unassigned) in the dataset. (b) Bar chart representing the normalized expression level of ACE2 protein across different cell types. (c) t-SNE plot highlighting ACE2 expression levels, with red dots indicating higher expression and green dots indicating lower expression. (d) InferCNV analysis for malignancy assessment, with a color gradient indicating expression levels and malignancy likelihood. ACE2: angiotensin-converting enzyme 2; t-SNE: t-distributed stochastic neighbor embedding; Tregs: regulatory T cells; CRC: colorectal cancer; NK: natural killer.

**Figure 2:**
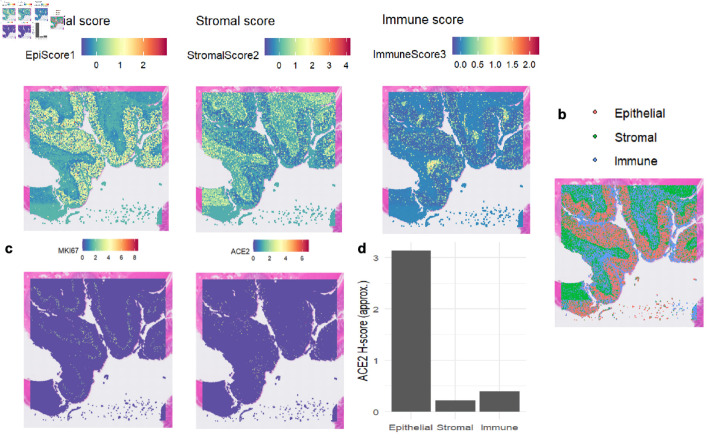
The expression levels of MKI67 and ACE2 proteins in colon tissue based on spatial transcriptomics. (a) Heat maps depicting regional scores of epithelial cells, stromal cells, and immune cells, illustrating the distribution of each component’s score. (b) Spatial distribution of epithelial cells, stromal cells, and immune cells, with different colors labeling the locations of the three cell components. (c) Heat maps showing the spatial distribution of *MKI67* and *ACE2* gene expression, where colors correspond to gene expression levels. (d) Bar chart of ACE2 H-scores in epithelial, stromal, and immune cells, comparing ACE2 expression levels across the three cell components. ACE2: angiotensin-converting enzyme 2; MKI67: marker of proliferation Ki-67.

Single-cell sequencing data indicated that ACE2 was highly expressed in malignant cells, with expression levels approaching 3, as shown in Figure 1a-c. InferCNV analysis confirmed the presence of diploid mutations in malignant cells, verifying their malignancy, as depicted in Figure 1d. The results of spatial deconvolution showed that three major cell regions could be clearly distinguished in the tissue sections: the epithelial regions, mainly distributed in the glands and the surface layer of the mucosa, exhibited the highest epithelial score; the stromal regions, located in the interstitial area of the glands, had a relatively high stromal score; and the immune regions, concentrated in the peripheral areas of the tissue or local infiltrative regions. Spatial gene expression analysis revealed that MKI67 was highly expressed in epithelial regions with active proliferation, while ACE2 was mainly concentrated in epithelial cell regions, and its expression was significantly reduced in stromal and immune regions. Further H-score analysis demonstrated that the average H-score of ACE2 in epithelial cells was approximately 3, which was much higher than that in stromal cells and immune cells (H-score < 0.5). This finding suggests that ACE2 expression has distinct cell-type specificity, being mainly restricted to epithelial cells, whereas only low-level background signals are observed in stromal and immune components, as illustrated in Figure 2.

### Upregulation of ACE2 expression in CRC tissues

**Figure 3:**
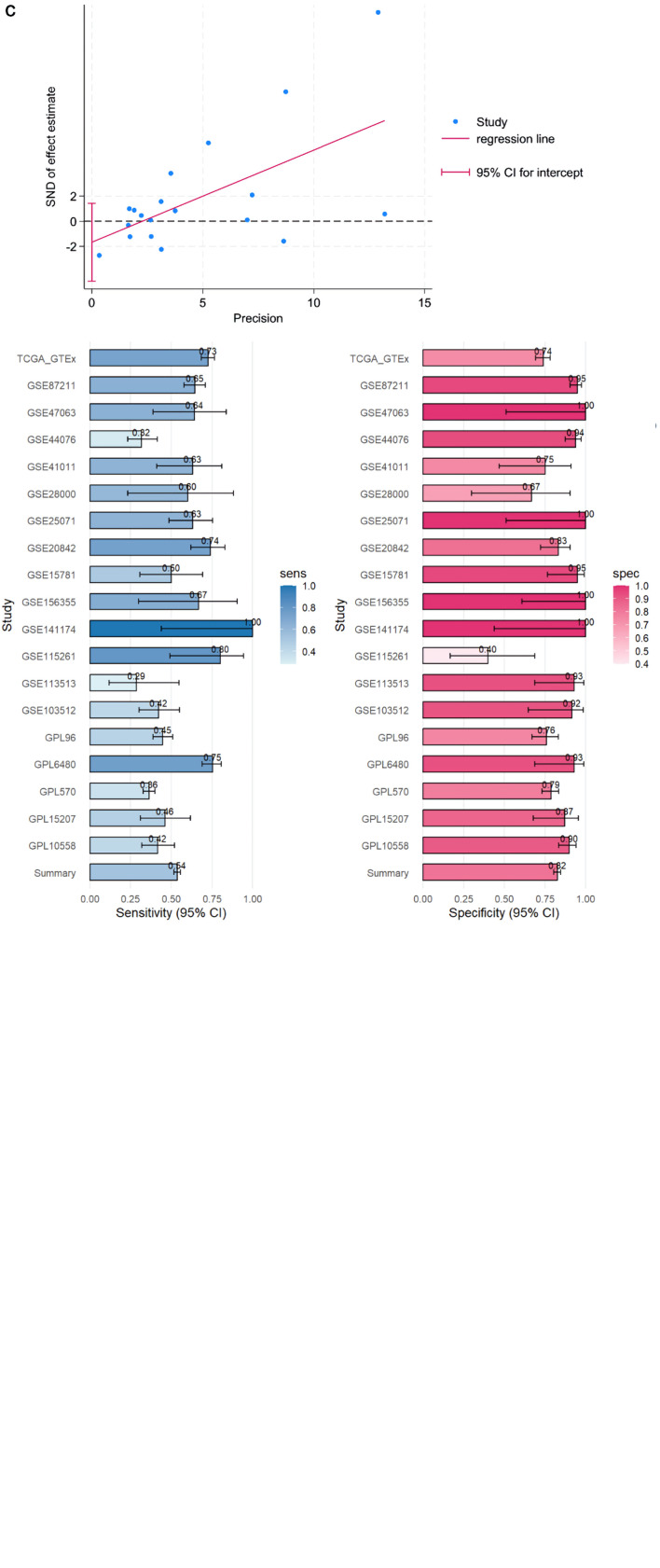
CRC ACE2 mRNA samples were obtained from a multicenter high-throughput dataset, including 2,275 CRC samples and 1,269 non-CRC colorectal samples. (a) Flow plot. (b) ACE2 expression in bulk mRNA data. (c) Egger’s test: detection of publication bias (P = 0.027); sensitivity and specificity analysis. ACE2: angiotensin-converting enzyme 2; AUC: area under the curve; CRC: colorectal cancer; GEO: Gene Expression Omnibus; TCGA: The Cancer Genome Atlas; SMD: standardized mean difference; SROC: summary receiver operating characteristic; CI: confidence interval; GTEx: Genotype Expression.

Multiple high-throughput datasets, comprising 2,275 CRC samples and 1,269 non-CRC colorectal samples, were used for the comprehensive analysis of ACE2 mRNA expression (Fig. 3a). A forest plot yielded an SMD of 0.321, indicating that ACE2 expression was significantly upregulated in CRC (Fig. 3b). The overall area under the SROC curve was 0.79 (95% CI: 0.75 - 0.82). The Begg’s test result indicated no publication bias (P > 0.05), and the funnel plot showed no heterogeneity (Fig. 3b). The integrated diagnostic efficacy of 19 studies showed a summary sensitivity of moderate level and a summary specificity of moderately high level (all studies in the forest plot largely overlapped with the overall estimate and were consistent in direction). The Deeks/Egger regression funnel plot showed no significant asymmetry, with the 95% CI of the intercept crossing zero, indicating no substantial small-study effects or publication bias. Sensitivity analyses were conducted, incorporating sequential leave-one-out exclusion of individual studies, application of continuity correction, and use of alternate estimation models. These analyses demonstrated no substantial changes in the direction or magnitude of pooled sensitivity and specificity, with overall significance remaining unaffected. Moreover, consistent conclusions were maintained even after removing outlier studies characterized by extreme sensitivity or specificity values. In summary, the sensitivity analysis confirmed that the combined estimate of diagnostic accuracy in this study is robust (Fig. 3c).

**Figure 4:**
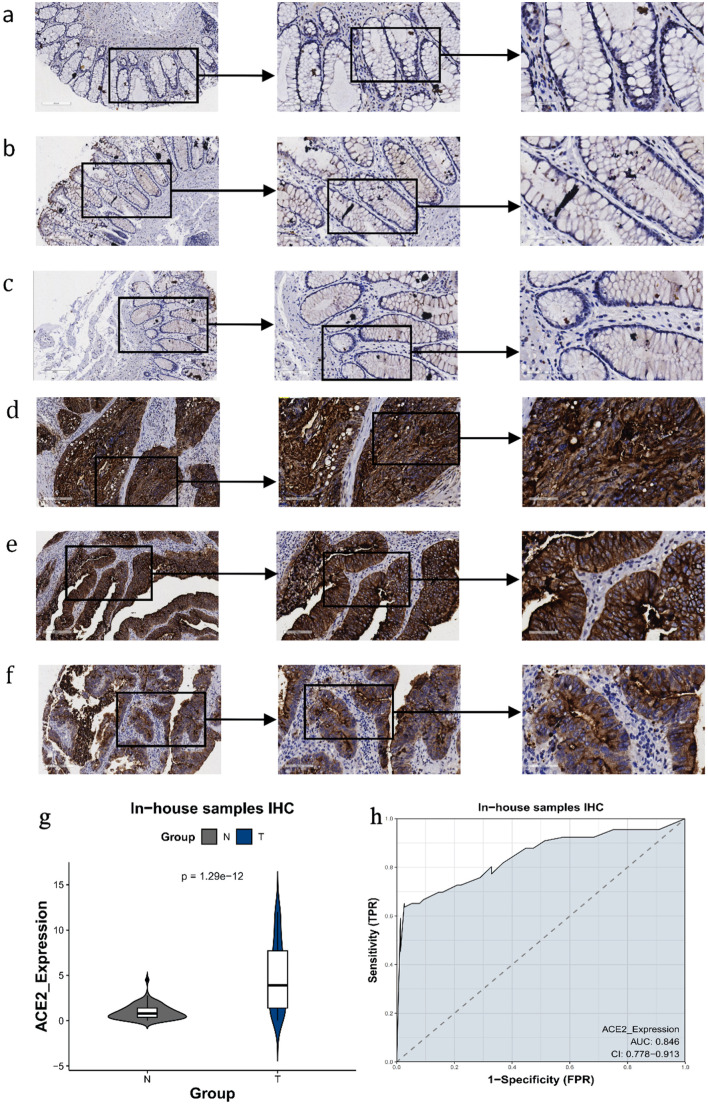
The expression level of ACE2 protein in CRC and peritumor colon tissues based on IHC. (a-c) Representative images of ACE2 protein expression in peritumor colon tissues. (d-f) Representative images of ACE2 protein expression in CRC tissues. In panels (a-f), the magnifications of the three images of each panel are 100, 200, and 400 respectively. (g) ACE2 protein expression (P = 1.29 × 10^-12^). (h) Receiver operating characteristic (ROC) curve with area under the curve (AUC) of ACE2 protein expression in CRC tissues. ACE2: angiotensin-converting enzyme 2; CRC: colorectal cancer; IHC: immunohistochemistry; TPR: true positive rate; FPR: false positive rate.

Compared to the non-CRC colorectal samples, the expression of the ACE2 protein in the CRC samples was significantly increased (Fig. 4). Positive staining signals for ACE2 were localized predominantly in the cytoplasm of CRC cells, rather than in paracancerous colonic mucosal cells. The AUC was 0.844, indicating a substantial discriminatory capacity of ACE2 expression in distinguishing between CRC samples and non-CRC colorectal samples.

### Other clinical value of ACE2 in CRC

**Figure 5:**
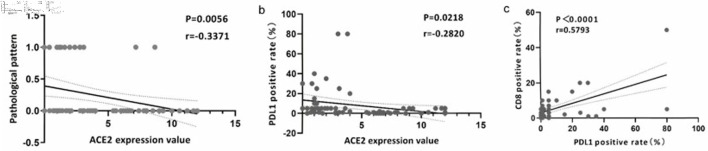
The expression of ACE2 was associated with CD8 and PD-L1. (a) Correlation analysis of ACE2 expression value and pathological pattern. (b) Correlation analysis of ACE2 expression value and PD-L1 positive rate (%). (c) Correlation analysis of PD-L1 and CD8 positive rate (%). ACE2: angiotensin-converting enzyme 2; PD-L1: programmed death ligand 1.

In the IHC analysis using internal samples (Table 2), we observed that CRC cases with nerve invasion exhibited elevated ACE2 protein expression levels, whereas mucinous adenocarcinomas generally displayed low or negligible ACE2 protein expression. Notably, there was a significant difference but no correlation between ACE2 expression values and nerve invasion. However, we observed a negative correlation between ACE2 expression values and pathologic types (r = -0.3371, P = 0.0056) (Fig. 5a). The remaining clinical parameters did not demonstrate statistical significance.

**Table 2:** The Relationships of ACE2 Expression With the Clinicopathologic Parameters by Interpretation of the Immunohistochemistry

Clinicopathological features	ACE2 expression				
	N	Mean	SD	P (t)	t/F
Tissue					
Non-cancer	75	0.973	0.803	< 0.001	-8.262
Cancer	66	4.917	3.804		
Gender					
Male	34	4.069	3.756	0.547	-0.675
Female	32	5.244	3.886		
Age					
< 60 years	16	3.769	3.685	0.594	-1.322
≥ 60 years	46	5.235	3.865		
Tumor stage					
T1 - T2	7	5.629	4.129	0.924	0.521
T3 - T4	59	4.832	3.792		
Node stage					
N0	42	5.224	3.773	0.993	0.866
N1 - N2	24	4.379	3.877		
Metastasis stage					
M0	62	5.024	3.719	0.441	0.903
M1	4	3.25	5.318		
Pathological stage					
Stage I - II	56	5.155	3.805	0.527	1.211
Stage III - IV	10	3.58	3.699		
Pathological type					
Tubular	52	5.577	3.832	0.008	3.603
Mucinous	14	2.464	2.548		
Vascular invasion					
No	59	4.59	3.689	0.395	-2.078
Yes	7	7.671	3.907		
Neurological invasion					
No	59	4.649	3.848	0.033	-2.265
Yes	7	7.171	2.632		
Tumor nodule					
Single	58	4.978	3.846	0.824	0.171
Multiple	7	4.714	3.917		
Tumor size (cm)					
< 5	32	5.216	3.567	0.374	0.617
≥ 5	34	4.635	4.047		
*BRAF* mutation (V600E)					
No	61	5.095	3.846	0.066	1.339
Yes	5	2.74	2.599		
*KRAS* mutation					
No	44	4.739	3.919	0.522	-0.535
Yes	22	5.273	3.623		
*NRAS* mutation (G12C/D/S)					
No	63	4.881	3.801	0.938	-0.347
Yes	3	5.667	4.623		
*NRAS* mutation (Q61R/L/H/K)					
No	62	5.094	3.837	0.04	2.785
Yes	4	2.175	1.855		

*BRAF*: B-Raf proto-oncogene, serine/threonine kinase; *KRAS*: Kirsten rat sarcoma viral oncogene homolog; *NRAS*: neuroblastoma RAS viral oncogene homolog; ACE2: angiotensin-converting enzyme 2; SD: standard deviation; P (t): P value from *t*-test; t/F: *t*-statistic/F-statistic.

### Relationship between ACE2 expression and *NRAS* mutation

In IHC and genetic test analyses conducted on internal samples (Table 2), we observed a significant difference in ACE2 expression values compared to the *NRAS* (Q61R/L/H/K) mutation, although no correlation was found. However, the remaining mutations did not exhibit statistical significance, including *BRAF* (V600E), *KRAS*, and *NRAS* (G12C/D/S).

### Correlation of ACE2 expression with T-cell subtypes

**Figure 6:**
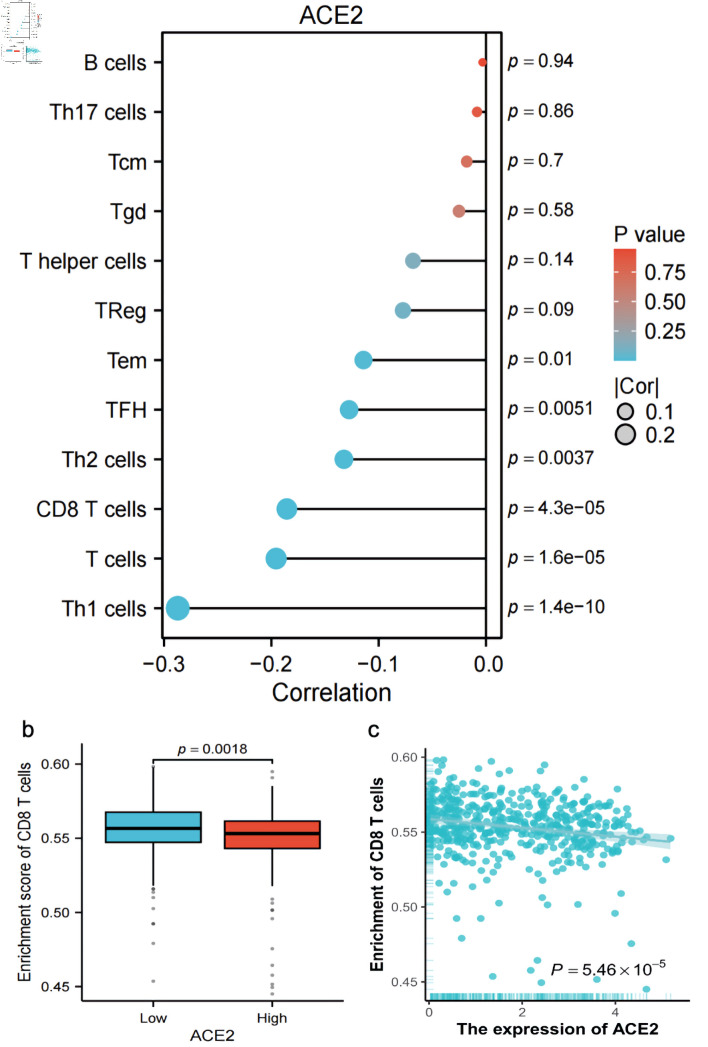
Correlation and enrichment analysis of ACE2 expression with immune cell types and CD8 T cell activity. (a) Bar plot showing the correlation (R) and P value of ACE2 expression with various immune cell types (Th1 cells, T cells, CD8 T cells, Th2 cells, TFH, Tem, Treg, T helper cells, Tgd, Tcm, Th17 cells, B cells), with color indicating P value significance. (b) Box plot comparing the enrichment score of CD8 T cells between low and high ACE2 expression groups (P = 0.0018). (c) Scatter plot showing the relationship between ACE2 expression (Log_2_(TPM + 1)) and CD8 T-cell enrichment score, with Spearman correlation (R = -0.186, P = 5.46 × 10^-5^). ACE2: angiotensin-converting enzyme 2; Tcm: T central memory; Tem: T effector memory; TFH: T follicular helper; Tgd: T gamma delta; Th1: T helper 1; Th17: T helper 17; Th2: T helper 2; Tregs: regulatory T cells; TPM: transcripts per million.

The expression of ACE2 exhibited a negative correlation with numerous T-cell subtypes, with correlation coefficients less than 0.1 and P values equal to 1.4 × 10^-10^, indicating a statistically significant inverse relationship (Fig. 6a). Additionally, a negative Spearman correlation (r = -0.186, P = 5.46 × 10^-5^) between ACE2 expression (log_2_(TPM + 1)) and CD8 T-cell enrichment was observed (Fig. 6b, c), suggesting that higher ACE2 expression was associated with reduced CD8 T-cell infiltration across the analyzed samples.

### Association of ACE2 expression with CD8 and PD-L1

In the IHC analysis based on internal samples, a negative correlation was observed between ACE2 expression levels and PD-L1 positivity (r = -0.282, P = 0.022) (Fig. 5b). Conversely, a positive correlation was found between CD8 positivity and PD-L1 positivity (r = 0.5793, P < 0.0001) (Fig. 5c).

## Discussion

Growing evidence links RAS to tumor growth and development [40-43]. ACE2, a key RAS regulator, has been well studied in COVID-19, but its role in CRC is unclear. This study analyzed 3,101 samples via multiple statistical methods and found significantly elevated ACE2 mRNA and protein levels in CRC. ACE2 protein expression in CRC patients correlated with tumor pathological type and PD-L1 expression and differed significantly in cases with neural invasion or NRAS (Q61R/L/H/K). These findings highlight ACE2’s high expression in CRC and its potential clinical significance and contribute to a deeper understanding of CRC’s molecular mechanisms.

Numerous studies have shown variable ACE2 expressions across tumor types. For example, it is downregulated in hepatocellular [38], breast [44], non-small cell lung [45], pancreatic ductal [46], and gallbladder cancers [47], but upregulated in endometrial and renal papillary cell carcinomas [48]. Previous CRC studies identified increased ACE2 [36, 49] but had limitations, such as small sample sizes (n < 500), limited data, or a lack of comprehensive multilevel analyses; some studies found no significant ACE2 protein differences. To address these gaps, our study used 66 in-house CRC samples, 76 non-CRC colorectal samples, and 2,960 multisource samples. We confirmed significant ACE2 upregulation in CRC (mRNA and protein levels). Importantly, ACE2 expression distinguished CRC from non-CRC samples - an unreported finding - and correlated with CRC tumor type.

Our study also found that colonic mucinous adenocarcinomas often showed low or no ACE2 expression. As a distinct subtype of CRC with specific molecular features [50, 51], this carcinoma progresses faster and is often diagnosed at an advanced stage compared to tubular adenocarcinoma [52]. Numerous studies have found that high Ang-(1-7) expression inhibits tumor proliferation, invasion, and migration in cancers such as nasopharyngeal [53], prostate [54], and lung cancer [55]. ACE2 has also been shown to inhibit tumor cell growth, metastasis, and angiogenesis in laryngeal, lung, gallbladder, and pancreatic cancers, as well as osteosarcoma [56]. Thus, low ACE2 expression is often linked to tumor progression. For example, downregulation of the ACE2/Ang-(1-7)/Mas axis promotes breast cancer metastasis by activating the Store-Operated Calcium Entry (SOCE) and p21-activated kinase (PAK)/nuclear factor kappa-light-chain-enhancer of activated B cells (NF-κB)/Snail1 pathways [57]. Notably, right-sided colonic mucinous adenocarcinoma correlates with extracellular matrix remodeling, EMT, and interleukin (IL)-17 signaling, while that on the left side may downregulate the ACE2/Ang-(1-7)/Mas axis via differentially expressed proteins (reducing ACE2 and Ang-(1-7)), potentially promoting tumor progression and metastasis [58]. This axis partially explains the low ACE2 expression in the carcinoma.

In addition, this study observed a negative correlation between ACE2 expression values and PD-L1 positivity, despite the small values of the correlation coefficient, and subsequent analysis revealed a positive correlation between PD-L1 positivity and CD8 positivity. Given that PD-L1 is a key regulator of CD8^+^ T-cell function [59], this finding suggests that ACE2 may indirectly influence T-cell infiltration and activity. Evidence indicates that ACE2 is negatively correlated with angiogenesis and the TGF-β signaling pathway [60], both of which have been demonstrated to induce PD-L1 expression [61]. Concurrently, ACE2 was positively correlated with an abundance of M2 macrophages [60], which can directly suppress CD8^+^ T-cell function and proliferation through the secretion of factors such as IL-10 and TGF-β [59]. However, it must be clearly stated that these findings, based on correlative analyses and pathway inferences, cannot establish a direct causal relationship between ACE2 and PD-L1 expression or CD8^+^ T-cell infiltration. Chronic inflammation is widely recognized as a contributor to multiple stages of tumorigenesis and development, encompassing cell transformation, survival, proliferation, invasion, angiogenesis, and metastasis. Notably, patients diagnosed with chronic inflammatory bowel disease face a substantially elevated risk of developing CRC [10, 62, 63]. Cytotoxic CD8^+^ T cells play a pivotal role in eradicating intracellular infections and malignant cells [16, 64], exhibiting a remarkable capacity to selectively identify and eliminate cancer cells [62]. The overexpression of PD-L1 hampers the cytolytic activity of CD8^+^ T cells and diminishes T cell-mediated cytotoxicity, allowing the evasion of immune surveillance and substantially promoting tumorigenesis and tumor aggressiveness [63]. In the liver, chronic inflammation leads to the accumulation of immunoglobulin A (IgA)^+^ plasma cells that express PD-L1 and IL-10, resulting in the direct suppression of cytotoxic CD8^+^ T cells and subsequent suppression of antitumor immunity [65]. Hence, the restoration of functional activity in CD8^+^ T cells within tumors is of paramount importance for the efficient eradication of tumor cells. Notably, natural killer group 2A (NKG2A)^+^ CD8^+^ T cells have been shown to be enriched in CRC, and obstructing NKG2A (or its ligands) enhances the cytotoxic response of these T cells toward CRC cells [66]. Furthermore, a positive correlation has been observed between an inflammatory immune microenvironment, elevated PD-L1 expression, and the response to immune checkpoint inhibitors, and upregulation of ACE2 may serve as an indicative factor for increased responsiveness to immunotherapy [31]. Additional investigations have highlighted that bromelain and ficin possess not only anti-infection therapeutic applications but also dose-dependent inhibitory effects on the expression of ACE2. Consequently, this inhibition hinders the proliferation of CRC cells and induces their apoptotic pathways [67]. Moreover, emerging strategies in cancer therapy involve repurposing drugs as GLP-1-based therapies or targeting 20S proteasomes, as well as administering various natural compounds, such as hinokitiol, as prophylactic agents with immunomodulatory effects and positive impacts on cancer [68-70]. Hence, the involvement of ACE2 in CRC could potentially be associated with the immune response. Moreover, the integration of multiple strategies that target the immune system through ACE2, in conjunction with programmed cell death 1 (PD-1)/PD-L1 blockade-based therapy, may prove to be a highly significant combinatorial approach for future immunotherapy endeavors.

Furthermore, this investigation observed that CRC cases with neurological invasion generally exhibited elevated levels of ACE2 expression. However, due to the limited number of cases, the correlation between ACE2 expression and neurological invasion could not be conclusively established despite the observed differences. Interestingly, a separate study investigating COVID-19-related brainstem invasion reported that the gastrointestinal epithelium expresses ACE2 receptors to a greater extent than the lung [71]. It also revealed that novel coronaviruses can directly infect intestinal cells, efficiently replicate within them, and invade nerves, subsequently ascending to the central nervous system via entero-vagal afferents. These findings suggest an alternative pathophysiological mechanism [72]. The present study elucidated the mechanism underlying nerve invasion by enterocarcinoma cells with high ACE2 expression. However, further investigation is required to determine whether CRCs exhibiting elevated ACE2 expression are more prone to nerve invasion.

CRC exhibits a multistage pathogenesis, progressing from early developmental abnormalities to adenomatous polyps and eventually to invasive carcinoma. The emergence of carcinoma is contingent upon the progressive accumulation of alterations that promote tumor growth over time [73]. At the molecular level, the adenoma-carcinoma sequence pathway is characterized by an abundance of *KRAS* mutations and somatic copy number alterations. Conversely, the serrated lesion-carcinoma pathway is driven by promoter hypermethylation of genes subsequent to *BRAF* mutations [74]. The prevalence of *NRAS* mutations in CRC is lower than the prevalence of *KRAS* mutations. *NRAS* mutations typically arise at a later stage of tumor progression and are often observed either alone or in combination with a *KRAS* mutation during tumor recurrence [75]. These mutations result in *KRAS* wild-type tumors that exhibit resistance to anti-EGFR therapy [76]. Specifically, the *NRAS* (Q61K) mutation promotes anchor-dependent proliferation and tumorigenicity, resembling the characteristics driven by classical *KRAS* mutations. Conversely, NRAS (G12D) expression reduces proliferation and increases apoptosis [76]. To investigate the association between ACE2 and common gene mutations in CRC, we conducted genetic testing for *BRAF* (V600E), *KRAS*, *NRAS* (G12C/D/S), and *NRAS* (Q61R/L/H/K). The positive rates for these mutations were approximately 6%, 28%, 4%, and 5%, respectively, which aligns with the rates reported in the literature [77]. Regrettably, no statistically significant differences were observed except for a notable disparity between NRAS (Q61R/L/H/K) and ACE2 expression. Functional studies have demonstrated that NRAS (Q61R/L/H/K), by impairing GTP hydrolysis, constitutively activates the RAF-MEK-ERK and PI3K-AKT signaling pathways [77, 78], subsequently upregulating c-Myc [79] and promoting cellular transformation and proliferation. It is noteworthy that the PI3K-AKT pathway has been reported to positively regulate ACE2 expression [36, 80], suggesting that NRAS (Q61R/L/H/K) may co-modulate ACE2 expression through this pathway, indicating a functional coupling at the signaling level. Furthermore, ACE2 mitigates inflammatory responses by degrading Ang II [81], potentially fostering a favorable immune microenvironment for *NRAS*-mutant tumors. Although the aforementioned mechanisms may exhibit variations across some tumor subtypes, the current evidence does not indicate significant differences in such signaling interactions among different subtypes of CRC. A previous study identified a significant reduction in ACE2 mRNA expression in *KRAS*-mutant tumors. Furthermore, ACE2 expression displays a negative correlation with KRAS, NRAS, Harvey rat sarcoma viral oncogene homolog (HRAS), and mesenchymal-epithelial transition (MET) but not with the expression levels of EGFR, BRAF, and ALK [82]. However, there remains a dearth of molecular mechanism studies exploring the relationship between ACE2 and *BRAF* (V600E), *KRAS*, and *NRAS* genes.

### Strengths

Our study successfully validated the upregulation of ACE2 in CRC and investigated its potential clinical significance and underlying mechanisms. The overexpression of ACE2 holds promise as a potential biomarker for identifying CRC. Moreover, our findings suggest that ACE2 may be involved in CRC through antiangiogenic and immune response pathways, warranting further investigation. These findings contribute to our understanding of the role of ACE2 in CRC and provide a foundation for future research in this field.

### Limitations

This study has several limitations that must be acknowledged. First, it lacks prognostic analysis, which would have provided valuable insights into the potential clinical implications of the findings. Additionally, the absence of *in vitro* functional experiments limits our ability to elucidate the underlying mechanisms and validate the observed associations. In the IHC cohort, there was insufficient statistical power when comparing different subgroups. These limitations highlight the need for further research to address these aspects and enhance our understanding of the subject matter.

### Conclusions

ACE2 protein expression shows a close correlation with pathologic type and PD-L1 positivity among patients with CRC. The underlying molecular mechanisms of ACE2 may be related to antiangiogenesis and immune response.

## Data Availability

The data supporting the findings of this study are available from the corresponding author upon reasonable request.

## Abbreviations

ACE2: angiotensin-converting enzyme 2
CRC: colorectal cancer
GEO: Gene Expression Omnibus
TCGA: The Cancer Genome Atlas
IHC: immunohistochemistry
KRAS: Kirsten rat sarcoma viral oncogene homolog
NRAS: neuroblastoma RAS viral oncogene homolog
BRAF: B-Raf proto-oncogene, serine/threonine kinase
ssGSEA: single-sample gene set enrichment analysis
PD-L1: programmed death ligand 1
AUC: area under the curve
SMD: standardized mean difference
ROC: receiver operating characteristic
SROC: summary receiver operating characteristic
CI: confidence interval
RAS: renin-angiotensin system
Ang II: angiotensin II
Ang-(1-7): angiotensin 1-7
AT1R: angiotensin II type 1 receptor
EMT: epithelial-mesenchymal transition
GTEx: Genotype Expression
scRNA-seq: single-cell RNA sequencing
UMAP: Uniform Manifold Approximation and Projection
SPSS: Statistical Package for the Social Sciences
TCGA-COAD: The Cancer Genome Atlas-Colorectal Adenocarcinoma
RNA-seq: RNA sequencing
TPM: transcripts per million
aDC: activated dendritic cells
iDC: immature dendritic cells
pDC: plasmacytoid dendritic cells
Tcm: T central memory
Tem: T effector memory
TFH: T follicular helper
Tgd: T gamma delta
Th1: T helper 1
Th17: T helper 17
Th2: T helper 2
Tregs: regulatory T cells
NK: natural killer
COVID-19: coronavirus disease 2019
SARS-CoV-2: severe acute respiratory syndrome coronavirus 2
VEGF: vascular endothelial growth factor
EGFR: epidermal growth factor receptor
ALK: anaplastic lymphoma kinase
HRAS: Harvey rat sarcoma viral oncogene homolog
MET: mesenchymal-epithelial transition
IL-17: interleukin-17
IgA: immunoglobulin A
IL-10: interleukin-10
NKG2A: natural killer group 2A
mRNA: messenger ribonucleic acid
STAR: Spliced Transcripts Alignment to a Reference
GSVA: Gene Set Variation Analysis
TGF-β: transforming growth factor-beta
ArrayExpress: ArrayExpress database
SOCE: Store-Operated Calcium Entry
PAK: p21-activated kinase
NF-κB: nuclear factor kappa-light-chain-enhancer of activated B cells
t-SNE: t-distributed stochastic neighbor embedding
MKI67: marker of proliferation Ki-67
SD: standard deviation
P (t): P value from *t*-test
t/F: *t*-statistic/F-statistic
